# Water and Beverage Consumption among Children Aged 4–13 Years in Lebanon: Findings from a National Cross-Sectional Study

**DOI:** 10.3390/nu8090554

**Published:** 2016-09-08

**Authors:** Lamis Jomaa, Nahla Hwalla, Florence Constant, Farah Naja, Lara Nasreddine

**Affiliations:** 1 Department of Nutrition and Food Sciences, Faculty of Agricultural and Food Sciences, American University of Beirut, P.O. Box 11-0.236, Riad El Solh, Beirut 11072020, Lebanon; lj18@aub.edu.lb (L.J.); nahla@aub.edu.lb (N.H.); 2 Nestle Waters, 12 boulevard Garibaldi, Issy-les-Moulineaux, Paris 92130, France; Florence.Constant@waters.nestle.com

**Keywords:** water intake, beverage consumption, water adequacy, hydration, children, Lebanon

## Abstract

This study evaluates total water intake (TWI) from plain water, beverages and foods among Lebanese children and compares TWI to dietary reference intakes (DRIs). In a national cross-sectional survey, data on demographic, socioeconomic, anthropometric, and physical activity characteristics were obtained from 4 to 13-year-old children (*n* = 752). Food and beverage consumption patterns were assessed using a validated food-frequency questionnaire. TWI was estimated at 1651 mL/day, with beverages contributing 72% of the TWI compared to 28% from foods. Beverages with the highest contribution to TWI included plain water, fruit juice and soda. A significantly higher proportion of 9–13-year-old children failed to meet the DRIs compared to 4–8 years old (92%–98% vs. 74%). Gender differentials were observed with a significantly higher proportion of boys meeting the DRIs compared to girls. The water to energy ratio ranged between 0.84 and 0.87, which fell short of meeting the desirable recommendations. In addition, children from higher socioeconomic status had higher intakes of water from milk and bottled water, coupled with lower water intakes from sodas. The study findings show an alarming high proportion of Lebanese children failing to meet TWI recommendations, and call for culture-specific interventions to instill healthy fluid consumption patterns early in life.

## 1. Introduction

Water is quantitatively the most important nutrient, playing a critical role in maintaining adequate hydration status [1]. Hypohydration is recognized as a precipitating factor in a number of acute medical conditions [2]. Even short periods of fluid restriction, characterized by a loss of body mass of 1%–2%, may lead to increases in self-reported tiredness and headache and to reductions in the subjective perception of alertness and ability to concentrate [2]. In addition, recent studies suggest that changes of hydration status may affect cognitive performance in children, whereby improved hydration was associated with enhanced performance on cognitive tests such as the digit-span and pair-cancellation tasks and with improved short-term memory [3,4].

Children are amongst the population groups that are at particular risk of hypohydration and inadequate water intakes [2,5,6]. Despite its critical importance in health and nutrition, the array of available research that serves as a basis for assessing the adequacy of water intake, remains limited in comparison with most other nutrients [1]. Adequate intakes for water are defined based on: (1) observed water intakes in various population groups; (2) desirable water volumes per 1000 kcal; and (3) desirable osmolality values in urine. The Dietary Reference Intakes (DRIs) for Water and Electrolytes reported by the Institute of Medicine (IOM) have set the Adequate Intake (AI) for total daily water intake at 1.7 L/day in 4–8-year-old children, 2.1 L/day in 9–13-year-old girls and 2.4 L/day in 9–13-year-old boys [7]. The European Food Safety Authority (EFSA) established total water AI levels at slightly lower levels: 1.6 L/day for boys and girls aged 4–8 years, and at 1.9 L/day for girls and 2.1 L/day for boys aged 9–13 years [8]. The water-to-energy ratio is another proposed index of adequate hydration; an index that incorporates to some degree, body size or surface area, and activity [1,9]. Accordingly, the desirable total water intake (TWI) is estimated to range between 1.0 and 1.5 L per 1000 kcal in children, depending on activity levels and water losses [8,10].

The established DRI values are based on water obtained from plain drinking water (bottled or tap), water from other beverages, and water from foods (both intrinsic water in foods and water added during food preparations) [8,10,11]. The DRIs were set mostly to prevent the adverse effects of dehydration, but beyond issues of hydration, there is increasing interest in characterizing consumption patterns of plain water vs. water arising from other sources. Some epidemiological data suggest that water may have different metabolic effects when consumed alone rather than as a component of flavored, sweetened or caffeinated beverages, but available evidence remains inconclusive [12,13]. Drinking plain water instead of caloric beverages helps to reduce dietary energy density, and may contribute to the regulation of body weight [1,9,14]. Some studies have also suggested that consumption of plain water is associated with better diets and better health behaviors in youth [15]. Based on the 2010 National Youth Physical Activity and Nutrition Study, Park et al. showed that low water intake was associated with poor diet quality and physical inactivity amongst US adolescents [16].

Although beverage consumption patterns and their contribution to energy intake (EI) have been well documented in children, few studies have explored the consumption of plain water and the adequacy of TWI in this age group [9,11,17,18]. A study conducted on a national sample of 4–13-year-old children in the US, showed that plain water, tap and bottled, contributed 25%–30% of total dietary water and that more than 75% of children did not meet the DRIs for TWI [9]. The study by Vieux et al. (2016), on 4–13-year-old French children and the study by Piernas et al. (2014), on Mexican children have also shown that the contribution of plain water to TWI did not exceed 34% and that a high proportion of children did not meet the recommendations (71%–90%) [11,17]. With the exception of few studies reporting on the contribution of various beverages to EI or on the volume of ingested fluids [19,20], no studies have investigated TWI and its adequacy amongst children in the Middle-East and North Africa (MENA), a region that is characterized by a hot climate, a high prevalence of dietary inadequacies in children [21,22] and one of the highest burdens of pediatric overweight and obesity worldwide [22]. Based on a nationally representative survey conducted in 2015, the present study aims at: (1) assessing total dietary water intakes (from foods and beverages) amongst 4–13-year-old children in Lebanon, in comparison with the IOM and EFSA recommendations by gender and age (4–8 years and 9–13 years); (2) investigating the association of water intakes from various sources with demographic, socioeconomic, anthropometric, and physical activity characteristics; (3) estimating EI from beverage and food sources and determining the water per calorie ratio (L/1000 kcal), in relation to desirable values by gender and age; and (4) comparing water intake data presented in this study to those reported from other countries, on the same age group.

## 2. Methods

### 2.1. Study Population and Sampling Framework

Data for this study were drawn from a national cross-sectional study conducted among a representative sample of children (4–18 years) and their mothers in Lebanon. A stratified cluster sampling strategy was followed, whereby the strata were the six Lebanese governorates and the clusters were selected further at the level of districts. Each district was divided into clusters comprised of 100–150 households. Households constituted the primary sampling unit within this national study. Within the cluster, households were selected by systematic sampling, based on probability proportional to size technique using the Lebanese Central Administration of Statistics as a reference [23]. For a household to be eligible, children and their mothers had to be present at the time of the interview. Inclusion criteria for children included: (1) Lebanese nationality; (2) child’s age between 4 and 18 years; (2) not suffering from any chronic disease; and (3) not taking any medications that may interfere with his/her dietary intake or body weight. Of the 4076 households that were contacted, 3147 accepted to participate in the study (response rate = 77%). Of these, 3147 households, 1221 met the eligibility criteria and 1209 completed the study. The main reasons for refusing to participate in the study were time constraints and lack of interest.

For the purpose of the present study, data for children aged 4–13 years were considered (*n* = 752). Since the evaluation of the TWI amongst 4–13-year-old children and the identification of the proportion of children meeting (or failing to meet) the water intake recommendations were among the main objectives of the national study, sample size calculations were conducted as follows: a minimum of 638 participants were needed to provide 95% confidence interval to estimate a prevalence of water adequacy of 30% with ±3.5% variation in this age group. The estimate of children with adequate water intake levels (30%) for the 4–13-year-old age group used in the sample size calculations was based on results from previously conducted studies reporting water and beverage consumption patterns among a similar age group of children and adolescents in Mexico [11] and the United States [9].

Ethical approval for the study was obtained from the Institutional Review Board at the American University of Beirut. Written informed consents were obtained from all mothers prior to participation in the study. Written assents were also obtained from children aged 6 years and above.

### 2.2. Data Collection

The survey was conducted over approximately one calendar year, between December 2014 and November 2015, covering weekdays and weekends. Face-to-face interviews with children and their mothers were conducted within their household setting by trained dietitians using a multi-component questionnaire. The questionnaire included information on demographic and socioeconomic characteristics, anthropometric measurements, dietary intake, and physical activity levels of participating children. Mothers served as proxy respondents for children under the age of 10 years and the interviews lasted on average 45 min per household.

Demographic characteristics included sex and age of the child. Indicators of the household’s socioeconomic status (SES) included parents’ highest educational level (intermediate level or less, high school or technical diploma, university degree or more), employment status (employed or unemployed) and household’s income (reported as <1 million Lebanese pounds (LBP)—662 US dollars equivalent, 1–1,999,999 million LBP, and ≥2 million LBP). Additionally, the crowding index, a commonly used criterion to assess the socio-economic status of households, was calculated by dividing the number of persons living in the household over the number of rooms in the households (excluding bathrooms, kitchens and balconies) [24,25]. The questionnaire was designed by a panel of experts including scientists in the fields of epidemiology and nutrition. The questionnaire was used in previous studies conducted in Lebanon [26,27] and was pilot tested on 25 households at the start of the present study to ensure clarity of questions.

Anthropometric measurements were obtained from study participants by trained dietitians using standard techniques and equipment. Children were weighed on a digital scale to the nearest 0.1 kg wearing light clothing, while height was measured to the nearest 0.1 cm, without shoes. Waist circumference (WC) was measured to the nearest 0.1 cm using a calibrated plastic measuring tape at the level of the umbilicus to the nearest 0.1 cm, after normal expiration. All measurements were taken twice and the average of the 2 values was reported. Body Mass Index (BMI) was calculated by dividing the weight in kilograms over the height in meters squared. Using WHO growth charts and criteria (WHO Growth reference 2015), BMI-for-age z-scores were used to classify children as normal weight, overweight, or obese. For children under 5 years, normal weight was classified as BMI between −2 and +2 SD of the WHO growth standard median, overweight as BMI > +2 SD, and obese as BMI > +3 SD. For children 5–13 years in the study sample, normal weight was defined between −2 SD and +1 SD, overweight > +1 SD, and obese > +2 SD above the WHO growth standard median. The Waist to height ratio (WHtR) for abdominal obesity was calculated by dividing WC by height, both measured in centimeters [28]. The suggested cut-off point of ≥0.5 was used to identify children with elevated WHtR [28,29].

Physical activity level of children was assessed using a modified version of the Children and Youth Physical Activity Questionnaire [30]. Children were asked to recall activities that they participated in and their duration during the past week, including weekdays and weekends, and activities within the school as well extra-curricular activities. For the purpose of this study, children engaging in more than 420 minutes per week of moderate to vigorous activities were considered active and those below this cut-off were categorized as inactive [31,32].

### 2.3. Dietary Intake Assessment and Interpretation

Dietary intake data was collected by trained interviewers using a 187-item food frequency questionnaire (FFQ) that was previously validated among Lebanese children to assess habitual dietary intake over the past year [33]. The FFQ included foods and beverages commonly consumed in Lebanon with a particular focus on a variety of beverages, such as bottled and tap water, milk, sodas, diet drinks, fruit and vegetable juices, hot beverages (coffee and tea), alcoholic beverages, and sports and energy drinks.

For children < 10 years old, mothers as the main meal planners were the proxy respondents to complete the FFQ. Children aged 10–14 years were the main respondents, and their mothers were present at the time of the interview to assist in providing detailed description of foods consumed at home including recipes and portion sizes consumed by children. To assist children and their mothers when estimating the portions and amounts of food and beverages consumed and reported in the FFQs, household measures and two-dimensional portion size posters were used (Millen and Morgan, Nutrition Consulting Enterprises, Framingham, MA, United States). These previously validated visuals [34] have been well-accepted and commonly used in previous national studies conducted in Lebanon [21,35].

### 2.4. Water and Energy Intake and Beverage Classifications

Daily water and EI from all foods and beverages reported in the FFQ were computed using the food composition database of the Nutritionist Pro software (version 5.1.0, 2014, SR 24, First Data Bank, Nutritionist Pro, Axxya Systems, San Bruno, CA, USA). The food composition database within this software is based on the USDA nutrient database [36]; however it was further expanded by adding analyses of traditional Lebanese foods and recipes reported among participating children using local food composition databases [37].

The present study focused on TWI from all foods and beverages. Results were reported as mL of water content from all foods and beverages, foods only, beverages only, and from specific beverages. Beverages were classified into 8 main groups with subcategories as follows: (1) plain water (bottled and tap); (2) milk and milk alternatives (milk shakes, yoghurt—plain or flavored—and hot chocolate prepared with milk); (3) sodas (regular and diet); (4) fruit juices (fresh fruit juice—100% natural, bottled fruit juice (without sugar), and bottled fruit juice with sugar (fruit drink); (5) vegetable juice; (6) hot beverages (coffee and tea); (7) sports and energy drinks; and (8) alcoholic beverages. EI from beverages were evaluated for the same beverage categories.

TWI from all food and beverages (mL/day) were compared to the Institute of Medicine (IOM) and EFSA water intake recommendations for each age and gender group to assess the shortfall in water consumption and the proportion of children who met or failed to meet the adequate water intake levels [7]. Using the same calculated daily TWI, in conjunction with total EI; water per calorie ratio was calculated and presented as mean (L/1000 kcal) by age and gender.

Water intake data estimated by the present study were compared to those reported by studies conducted in other countries, on similar age groups. Dietary assessment methods varied across countries, with the 24-hour recall data being adopted in Mexico and the US [9,11], and the 7-day food record being used in France [17]. These studies reported on plain water (tap and bottled) and similar beverage groups, while also sharing similar definitions for the assessment of TWIs, water and energy intakes from foods and beverages and their contribution to TWI and EI [9,11,17]. The estimated TWIs were compared to the IOM water intake recommendations in Mexico and the US [9,11], whereas the study conducted in France [17] used the EFSA recommendations [17].

### 2.5. Statistical Analysis

Continuous variables were presented as means and standard errors (SE), whereas categorical variables were reported as proportions with percentages.

Mean TWI from different beverage groups and from moisture in foods were presented by age group (4–8 years and 9–13 years), gender, socioeconomic, anthropometric and physical activity characteristics. The contribution of water from each beverage type was calculated at the individual level by dividing the water intake from that specific beverage type by the daily TWI. Comparisons of mean daily TWIs and mean % of TWI from each beverage type were conducted by age group (4–8 vs. 9–13 years old) using student *t*-tests. Associations of water intakes (from foods and beverages; plain water, and beverages (excluding plain water) with demographic, socioeconomic, anthropometric, and physical activity characteristics, were examined using student *t*-tests and analysis of variance (ANOVAs) with Bonferroni corrections.

In addition, the mean daily intake of energy (kcal) from all foods and beverages, from caloric beverages, and from each beverage type were calculated and presented as means ± SE and as proportions (percent of total EI). Differences between 4–8- and 9–13-year-old children in terms of mean EI from each beverage type and the contribution of beverages to total EI were conducted using student *t*-tests. Using the IOM age and gender-specific water recommendations, the proportion of children who met or did not meet the DRIs for water intake, the total shortfall in water consumption, and mean ratio of water per calorie (L/1000 kcal) were calculated. Similar calculations were conducted to compare the average TWI of children in the study sample with EFSA water intake recommendations by age and gender.

All data analyses were conducted using the Statistical Package for the Social and Sciences statistical software package (SPSS) version 22 with *p*-values of < 0.05 considered statistically significant.

## 3. Results

### 3.1. Total Water Intakes among Study Sample in Relation to Recommended Intakes

Average TWI for all children (aged 4–13 years old) in the study sample was assessed to be 1651 mL/day. As shown in Table 1, average TWI was estimated at 1601 mL/day amongst 4–8-year-old children and 1698 mL/day amongst 9–13 years old. Overall, intakes of water from all foods and beverages, plain water and from beverages (excluding plain water) were significantly higher among boys than girls and among older (9–13 years) compared to younger children (4–8 years).

**Figure 1:**
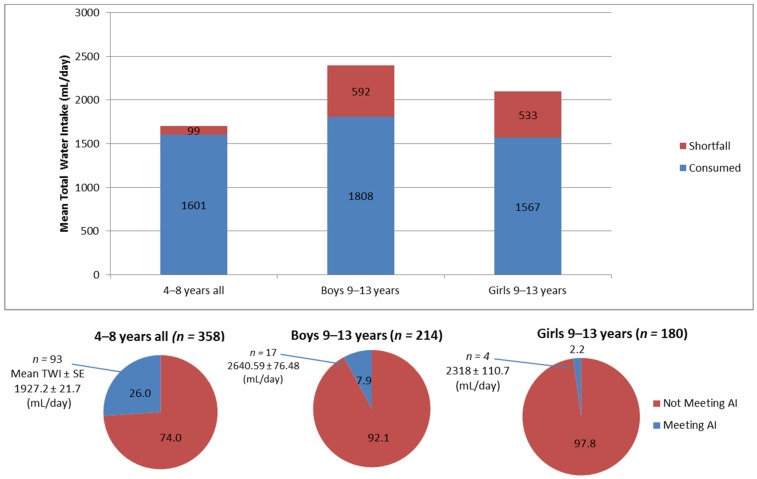
Shortfalls in total water intakes amongst 4–13-year-old Lebanese children as compared to the IOM adequate intake (AI) and proportion of children meeting the age and gender-specific AIs. Total daily water intake from all foods and beverages (consumed) by age group and gender compared to Institute of Medicine Recommendations (IOM) (shortfall). Proportions of children meeting and not meeting needs compared to IOM are displayed in the corresponding pie charts.

Total daily water intakes amongst 4–13-year-old Lebanese children were compared to the age and gender-specific water intake recommendations of the IOM (Figure 1). Compared to the AI values, the shortfall in water intake was observed to be higher amongst 9–13-year-old children (592 mL/day in boys and 533 mL/day in girls) compared to their younger counterparts (99 mL/day). Overall, only 15% (*n* = 114) of the study sample met the recommendations for daily TWI, among whom the mean TWI was estimated at 2047 mL/day (SE = 32.2) on average. In addition, a significantly higher proportion of 4–8-year-old children met the AI values compared to 9–13 years old (26% vs. 5%, *p* < 0.001). Amongst the older age group, a higher proportion of boys met the water intake recommendations compared to girls (7.9% vs. 2.2% *p* = 0.012). Similarly, a significantly higher proportion of boys had adequate levels of TWI compared to girls within the 4–8-year-old children group (41.5% vs. 9.7%), *p* < 0.001 (data not shown).

Additional analyses were conducted based on EFSA recommendations for adequate water intakes. Accordingly, 26.5% of 4–13-year-old children were found to meet the AI values. Similar to the observations seen with the IOM DRIs, a higher proportion of younger children met the EFSA water intake recommendations compared to older children (40.5% vs. 13.7%, *p* < 0.001) and a higher proportion of boys had adequate levels of TWI compared to girls, in both age groups (*p* < 0.001).

As for the water-to-energy ratio, the observed water volume per 1000 kcal ranged between 0.84 in 4–8-year-old children and 0.87 in 9–13 years old, with 96%–99% of the children not meeting the desirable IOM recommendations for this indicator.

### 3.2. Water Consumption According to Socioeconomic, Physical Activity and Anthropometric Characteristics

Water intakes were not found to be significantly different according to socioeconomic indicators (household income, crowding index, mother and father’s educational level or employment status) (Table 1). However, results showed that children reporting active physical activity levels had significantly higher TWI from foods and beverages and higher water consumption from beverages compared to inactive children. No significant associations were observed between various anthropometric measures (such as BMI and WHtR) with water consumption from total foods and beverages, plain water, and beverages (excluding plain water).

### 3.3. Patterns of Water Intake from Food and Various Beverage Types by Age and Gender

Table 2 presents the intakes of water from foods and from different beverage types by age group. Overall, water from beverages contributed close to 72% of total daily water intake amongst 4–13-year-old children, compared to 28% from food moisture. Amongst 4–8-year-old children, the three main sources of water included plain water (47.6%), moisture in foods (28.5%), and fruit juice (10.3%). For older children (9–13 years), the main contributors to TWI included plain water (49%), moisture in food (27.7%), and sodas (10.3%). Older children (9–13 years) were found to have significantly higher water intakes from plain water (bottle and tap water), sodas, particularly regular/caloric sodas, hot beverages, energy drinks and alcoholic beverages compared to younger ones (4–8 years). In addition, the contribution of water intake from plain water, sodas, and alcoholic beverages to TWI was significantly higher amongst 9–13-year-old children compared to their younger counterparts. On the other hand, the contribution of water intake from foods, milk and milk alternatives, and fruit juice (fresh and bottled with sugar) were significantly higher amongst 4-8-year-old children compared to the older ones.

**Figure 2:**
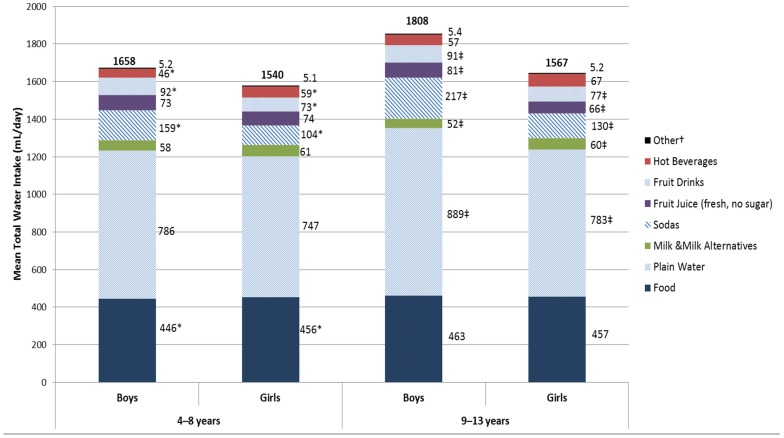
Total daily water intake (mL/day) from all sources by gender, in a national sample of 4–13-year-old children. * Statistical comparisons are made between genders within the 4–8 years age group, derived from independent samples *t*-test, results are significant at *p* < 0.05. ‡ Statistical comparisons are made between genders within the 9–13 years age group, derived from independent samples *t*-test, results are significant at *p* < 0.05. † Other category includes: vegetable juices, sports and energy drinks, and alcoholic beverages.

Gender differentials in water intakes from foods and from different beverage sources are shown in Figure 2. For the 4–8-year-old children, water intakes from solid foods and from hot beverages (tea/coffee) were found to be significantly higher amongst girls compared to boys. On the other hand, 4–8-year-old boys had significantly higher intakes of water from sodas and fruit drinks (bottled fruit juices with sugar) compared to girls of the same age. Among 9–13-year-old children, significantly higher water intakes from sodas, fruit drinks, and fruit juices (fresh and no sugar added) were noted in boys compared to girls, whereas significantly higher water intakes from milk and milk alternatives were observed in girls compared to boys.

### 3.4. Water Intake from Specific Beverages by Socioeconomic, Anthropometric and Physical Activity Characteristics

Table 3 presents the intakes of water from specific beverages by socioeconomic, anthropometric, and physical activity characteristics amongst 4–13-year-old Lebanese children. A higher paternal education level (university degree or more) was found to be associated with higher water intakes from bottled water, coupled to lower intakes from tap water and regular soft drinks. As for maternal educational level, it was found to be associated with higher intakes of bottled water, milk and milk alternatives yet with lower intakes of tap water, regular soft drinks and energy drinks. Monthly family income and crowding index, two indicators of socioeconomic status, were also found to be associated with water intakes from specific beverages: Children from households with higher socioeconomic status had higher intakes of water from bottled water, milk and milk alternatives, fresh fruit juice and vegetable juice, yet lower intakes of water from tap water and caloric soft drinks. With respect to physical activity, overall active children had higher intakes of water from beverages compared to inactive ones, with the difference reaching statistical significance for soft drinks and fresh fruit juice. No significant associations were observed between anthropometric measures, such as BMI status and WHtR, with water intakes from plain or bottled water, milk, soft drinks, and juices (data not shown).

### 3.5. Contribution of Various Beverage Sources to Total Daily Energy Intake

As shown in Table 4, on average, older children (9–13 years) had a significantly higher EI from all foods and beverages, foods only, and caloric beverages compared to younger ones (4–8 years old). Foods were found to be the main contributors to EI in both age groups (90.5%–90.6% EI), whereas beverages contributed close to 9% of the daily caloric intake. Overall, beverages with the highest contribution to EI of children aged 4–13 years old included fruit juices (mainly fruit drinks), sodas (regular), and milk and milk alternatives. Compared to their younger counterparts, older children (9–13 years) had significantly higher EIs and higher contribution of specific beverages to total daily EI, including regular sodas, hot beverages, and energy drinks. On the other hand, we observed that the contribution of milk and milk alternatives and fruit juices to total daily EI was significantly higher amongst younger compared to older children (*p* < 0.05).

### 3.6. Comparison of Water Intakes amongst Children in Lebanon with Other Countries

Table 5 compares the results of this study to those reported from other countries, on the same age group [11,17,38]. Accordingly, TWI amongst 4–8-year-old Lebanese children (1600 mL/day) exceeded that reported for French, American and Mexican children (1233–1447 mL/day), while the intake amongst 9–13 years old was within the range reported from other countries. Water intakes from food sources in Lebanon (450–460 mL/day) were inferior to those reported from Mexico (505–597 mL/day) and France (492–555 mL/day), while the consumption of plain water was the highest in Lebanon (767–840 mL/day), exceeding intake levels reported by other studies (350–498 mL/day). Milk’s contribution to TWI was low in Lebanon (3.4%), compared to estimates reported by France and the USA (13%–20%). Conversely, soda was identified as one of the three main contributors to water intake in Lebanese children (8%–10%).

## 4. Discussion

Based on a nationally representative survey, this study explored total water intake amongst 4–13-year-old children in Lebanon, in comparison to international recommendations, making it the first to report on the intake of this critical nutrient among children from the MENA region. Studies of beverage consumption amongst children have mostly focused on caloric beverages such as milk [39,40], fruit juices [41,42], sweetened beverages [40,42,43,44,45], and the amount of dietary energy provided in liquid form [17]. Few studies have investigated patterns of water consumption by age group, sex and socioeconomic status, and even fewer studies have compared water intakes amongst children with the existing recommendations and across various countries [9,11,17,18].

This study showed, in agreement with findings reported by other studies [9,11,17], that total daily water intakes amongst 4–13-year-old Lebanese children were below the existing recommendations. When comparing age groups, the study results indicated that a higher proportion of older children failed to meet the IOM DRI values compared to their younger counterparts (4–8 years old). The shortfalls in total daily water intakes, based on the IOM DRI values, were the highest amongst 9–13-year-old children partly because the AI for water increases with age [46]. Data were reanalyzed using the EFSA AI values and the same observations were noted. When comparing our results to those derived by other studies, Lebanon had the highest proportion of 9–13 years old who did not meet the water intake recommendations, followed by France (90%–93%), USA (83%–85%) and Mexico (81%–83%) [9,11,17]. Gender differentials in water intake were also documented in the present study, with a significantly higher TWI observed amongst boys and a significantly higher proportion of boys meeting the AI values compared to girls. The observed gender differentials in water intakes may be a reflection of the significantly higher proportion of boys engaging in physical activity compared to girls in our study sample (66% vs. 49%, *p* < 0.001, data not shown), and therefore of their higher water needs. As for the water to energy ratio, another indicator of hydration, the study findings showed that the water volume per 1000 kcal ranged between 0.84 and 0.87, which fell short of meeting the desirable IOM recommendations of at least 1 L/1000 kcal. Taken together, the study’s findings highlight a substantial gap between existing recommendations and actual water intakes in Lebanese children. This is of concern given the vulnerability of this age group to dehydration and its adverse effects. Children have a higher proportion of body water compared to adults; they are also less heat tolerant and more susceptible to dehydration, especially in hot climates and when engaging in physical activity [1,47]. Available studies suggest that low to moderate levels of dehydration may increase fatigue, decrease alertness and impair cognitive function, which may carry implications on school performance [1,3,4,48,49].

The high proportion of children not meeting the international water intake recommendations in Lebanon as well as in other countries may be at least partly explained by the fact that water consumption tends to be underreported, particularly among children [11,38]. This highlights the need for rigorous investigations on probing methods to collect better water recall data and reduce measurement errors, mainly amongst the pediatric population [1]. It is also important to underscore the scarcity of available research serving as evidence for setting adequate water intake levels, despite the critical importance of water in health and nutrition. While this scarcity may be partially explained by the sophisticated set of neurophysiological adaptations and adjustments that occur over a large range of fluid intake to protect body hydration and osmolality, it remains a challenge for nutrition and public health professionals [1]. In addition, given the extreme inter-individual variability in water needs that are not only determined by differences in metabolism, but also by environmental conditions and physical activities, there may not be a single level of water intake that would assure adequate hydration [1]. Water needs may in fact be influenced by the individual’s health status, physical activity, dietary intake, including sodium and protein intake, and environmental factors such as temperature and humidity [38].

In order to better contextualize the observed water intake data, the results of this study were compared to those reported from other countries, for the same age group [9,11,17]. The consumption of plain water was found to be the highest in Lebanon exceeding intake levels reported by other studies and contributing to almost half of TWI. Even though milk appeared as one of the main contributors to TWI in France and the USA (13%–20%), milk’s contribution to TWI was low in Lebanon, being estimated at 3.5% in the study sample. On the other hand, soda was identified as one of the three main contributors to water intake in Lebanese children, even amongst the younger age group. Water intakes from food sources were lower than those reported from Mexico and France [11,17], suggesting a lower consumption of low-energy-density foods, such as fruits and vegetables, amongst Lebanese children. For instance, the intake of fruits and vegetables was previously estimated at 87 g/d amongst Lebanese children aged 6–11 years [22], compared to estimates ranging between 141–152 g/day amongst 3–14-year-old French children [50].

The present study has also investigated the association between water intakes and SES. Interestingly, and in contrast to data reported from the USA and Mexico [9,11,38], this study’s findings showed that total and plain water intakes were not associated with the household’s income. When examining the association between SES and water intakes from specific beverage sources, a higher SES was found to be associated with a healthier pattern of beverage consumption. Several SES indicators, including paternal and maternal education levels, family income and crowding index, converged in showing that water intakes from bottled water increased with increasing SES, while the opposite was observed for sodas and tap water. Available evidence suggests that in Lebanon, tap water may not be safe for consumption as its quality tends to deteriorate during distribution, namely due to cross-contamination by wastewater networks and rusting water conduits [51]. This may explain the observed association between higher SES and bottled water consumption, as households who can afford it will naturally opt for safer sources of water. Higher maternal education was associated with a healthier drinking pattern amongst Lebanese children, characterized by higher water intakes from milk, and lower intakes from soft drinks and energy drinks. These findings are in line with those reported by other studies, where soft drink consumption, dairy product intakes and dietary adequacy amongst children were found to be directly related to maternal education levels [52,53,54]. This highlights the role of the mother’s education and awareness in modulating the family environment, which can have a direct influence on the child’s lifestyle and dietary behavior, including fluid consumption [52,53,54].

In this study, physically active children had higher intakes of total water, which is expected given that their hydration needs are higher. Physically active children were also found to have lower intakes of sodas and higher intakes of fresh fruit juice (no added sugar). These findings are aligned with those reported amongst a national sample of Lebanese adolescents aged 13–19 years, where physically active children were found to follow a healthier dietary pattern compared to those with low levels of physical activity. These observations may suggest that the clustering of behavioral risk factors, including physical inactivity and unbalanced diet, which has been repeatedly described among adults [55], is already apparent as early as the childhood and adolescent years [21]. Even though the present study’s findings did not document a protective association of TWI or plain water intake against overweight and obesity, other studies have suggested that higher water intakes may be associated with healthier weight in children [1,14]. This may be of direct relevance to the MENA region that harbors one of the highest rates of childhood obesity in the world. In Lebanon, pediatric obesity is following an alarming escalating trend over time, with the prevalence of obesity increasing from 7.3% to 10.9% in 6–19-year-old children and adolescents, over the past decade [56].

The present study showed that the contribution of beverages to daily dietary EI was close to 9% amongst Lebanese children, which is lower than estimates observed in other countries (10%–20%) [9,11,17]. However, unlike data reported from other studies where milk appeared as the beverage with the highest contribution to EI (7%–11%), Lebanese children’s milk intake contributed less than 3% to daily EI. At the same time, soda was identified as the beverage with the second most important contribution to EI in both age groups in Lebanon. These findings suggest that soda consumption may be displacing milk drinking in Lebanese children, which could result in decreased calcium intakes, suboptimal bone health [57], disrupted calcium-phosphorus ratio [58,59], overweight and obesity [60,61].

The strengths of this study include the national representativeness of the sample, the use of a validated FFQ in dietary assessment, and the measurements of anthropometric characteristics by trained dietitians instead of self-reporting. The age cut-off points that were selected for this study (4–8 years and 9–13 years) were intentionally similar to those adopted by the IOM and by EFSA when setting DRIs for children, allowing for direct comparisons with the AI values. The results of the present study should, however, be considered in light of the following limitations. Dietary intake data were collected by means of a FFQ that may be subject to respondent and recall bias. Proxy recall for younger children may represent an additional source of bias. Recall bias may be particularly challenging when exploring water intake among children, who may consume it unconsciously during their regular day (within the school meal, when playing in public playgrounds, during social events, and other venues) [9,11]. In addition, there was no measurement of hydration status validating reported TWI in the present study. Another limitation is the lack of data on the time and occasions of water and beverage consumption throughout the regular day (breakfast vs. lunch vs. dinner and snacking patterns). Future studies need to explore potential differences in water and beverage consumption patterns across various occasions, days of the week, and time of day. Finally, the cross-sectional design of the study allowed us to test associations rather than to assess any causal relationships.

## 5. Conclusions

In conclusion, even though the intakes of plain water in Lebanon were higher than those reported by other countries, and the contribution of caloric beverages to EI were lower than that observed in other countries, the results of this study showed that an alarming high proportion of Lebanese children failed to meet water intake recommendations. In addition, older children aged 9–13 years, and girls were identified by this study as being at particular risk of water intake inadequacies. These findings raise a public health concern, given the vulnerability of children to dehydration and its potential adverse effects [1]. The study’s findings have also shown that, compared to children from high SES, children from a lower socioeconomic background had higher water intakes from soda, energy drinks and tap water coupled with lower water intakes from milk, fresh fruit juice, vegetable juice and bottled water. This socioeconomic gradient should be taken into consideration when planning for intervention strategies aiming at instilling healthy drinking habits at an early age in Lebanon. Future dietary guidelines and policy interventions should promote drinking plain water for daily hydration and nutrient-dense beverages, such as low-fat milk, to contribute further to adequate fluid intake and nutrient adequacy amongst children. This is of particular importance given the magnitude of the shortfall between observed TWI and DRIs. A particular challenge would be the safety of tap water in Lebanon, given that surveillance measures and water quality monitoring are lagging behind in the country. Schools should be encouraged to make safe potable water available to all students, by installing fresh water fountains and providing children with easy access to a non-caloric beverage at no charge [9,17]. Along with improving the availability of safe water, it is crucial to decrease students’ access to caloric beverages in the school setting, by limiting the sale and marketing of sugar-sweetened beverages. Supportive educational programs targeting students and parents, as agents of change, are needed to foster healthy drinking patterns early in life [62].

## Figures and Tables

**Table 1:** Total Water Intakes (mL/day) from foods and beverages by sociodemographic, anthropometric and physical activity characteristics in a representative sample of Lebanese children aged 4–13 years (*n* = 752).

	Total Mean ± SE or *n* (%)	TWI from Foods and Beverages (mL/Day)	Water Intakes from All Beverages (mL/Day)	
			Plain Water (Bottled and Tap) (mL/Day)	Beverages (Excluding Plain Water) (mL/Day)
	Mean ± SE			
**Age**	9.07 ± 0.10			
4–8 years	358 (47.6)	1600.63 ± 12.93 ^a^	767.32 ± 11.22 ^a^	376.21 ± 7.95 ^a^
9–13 years	394 (52.4)	1697.68 ± 16.55 ^b^	840.49 ± 13.39 ^b^	401.13 ± 9.24 ^b^
**Gender**				
Boys	397 (52.8)	1738.77 ± 17.72 ^a^	841.58 ± 13.50 ^a^	440.54 ± 9.91 ^a^
Girls	355 (47.2)	1553.87 ± 8.81 ^b^	765.48 ± 10.97 ^b^	331.92 ± 5.47 ^b^
**Father’s educational level**				
Intermediate or less	435 (58.2)	1646.44 ± 13.60 ^a^	804.73 ± 11.53 ^a^	396.18 ± 7.49 ^a^
High school/technical diploma	203 (27.2)	1660.32 ± 22.73 ^a^	812.81 ± 18.60 ^a^	378.70 ± 14.37 ^a^
University Degree or more	109 (14.6)	1645.37 ± 27.34 ^a^	793.63 ± 21.15 ^a^	372.21 ± 12.91 ^a^
**Mother’s educational level**				
Intermediate or less	363 (48.3)	1632.03 ± 14.98 ^a^	790.21 ± 12.32 ^a^	391.70 ± 9.36 ^a^
High school/technical diploma	227 (30.2)	1674.59 ± 20.87 ^a^	830.57 ± 17.45 ^a^	393.16 ± 10.89 ^a^
University Degree or more	162 (21.5)	1662.68 ± 22.69 ^a^	805.37 ± 18.64 ^a^	378.35 ± 12.04 ^a^
**Father’s employment status**				
Unemployed	32 (4.3)	1669.58 ± 62.48 ^a^	794.76 ± 60.61 ^a^	436.67 ± 33.85 ^a^
Employed	711 (95.7)	1651.28 ± 11.01 ^a^	807.07 ± 8.99 ^a^	386.36 ± 6.28 ^a^
**Mother’s employment status**	743 (100)			
Unemployed	556 (74.0)	1644.78 ± 12.56 ^a^	802.36 ± 10.43 ^a^	388.29 ± 7.22 ^a^
Employed	195 (26.0)	1669.90 ± 21.04 ^a^	814.06 ± 17.21 ^a^	391.98 ± 11.88 ^a^
**Monthly family income (LBP)**				
<1,000,000	310 (41.8)	1636.68 ± 16.11 ^a^	807.52 ± 14.25 ^a^	391.02 ± 8.91 ^a^
1,000,000–1,999,999	279 (37.6)	1643.13 ± 16.75 ^a^	790.03 ± 14.02 ^a^	386.28 ± 9.05 ^a^
≥2,000,000	153 (20.6)	1701.01 ± 27.93 ^a^	835.94 ± 20.22 ^a^	391.31 ± 17.74 ^a^
**Crowding index (person/room)**	1.62 ± 0.03			
<2	534 (71.2)	1654.31 ± 13.01 ^a^	808.22 ± 10.58 ^a^	388.52 ± 7.50 ^a^
≥2	216 (28.8)	1644.11 ± 19.30 ^a^	799.48 ± 16.74 ^a^	390.62 ± 10.72 ^a^
**BMI z-score**	0.92 ± 0.06			
**BMI status**				
Normal weight	443 (58.9)	1640.08 ± 13.96 ^a^	787.19 ± 10.82 ^a^	390.21 ± 8.49 ^a^
Overweight	152 (20.2)	1657.23 ± 23.68 ^a^	824.72 ± 20.89 ^a^	376.22 ± 12.41 ^a^
Obese	157 (20.9)	1678.07 ± 24.21 ^a^	839.29 ± 21.70 ^a^	399.24 ± 12.33 ^a^
**Waist Circumference (cm)**	64.11 ± 0.46			
**Waist to height ratio (WHtR)**	0.48 ± 0.00			
<0.5	489 (65.4)	1660.89 ± 14.08 ^a^	807.59 ± 242.03 ^a^	392.54 ± 8.17 ^a^
≥0.5	259 (34.6)	1631.77 ± 260.18 ^a^	803.33 ± 250.37 ^a^	382.24 ± 144.39 ^a^
**Level of physical activity**				
Active	434 (57.7)	1676.47 ± 13.94 ^a^	817.52 ± 12.16 ^a^	404.27 ± 7.39 ^a^
Inactive	318 (42.3)	1617.37 ± 16.78 ^b^	789.46 ± 12.95 ^a^	368.80 ± 10.41 ^b^

^a,b^ Statistical comparisons were conducted within each sociodemographic group and within anthropometric and physical activity levels based on independent samples *t*-test or ANOVA test. Mean estimates with different superscript letters are significantly different at *p* ≤ 0.05.

**Table 2:** Intakes of water (mL/day) from different beverage groups and from foods and their contribution to total water intake by age group; children aged 4–13 years, Lebanon (*n* = 752).

	Total 752		4–8 Years 183 (46.1%)		9–13 Years 214 (53.9%)		*p*-Value
	Mean ± SE	% of TWI	Mean ± SE	% of TWI	Mean ± SE	% of TWI †	
**Total water intake from all food and beverages**	1651.48 ± 10.77	100	1600.63 ± 12.93 ^a^	100	1697.68 ± 16.55 ^b^	100	*p* < 0.001
**Water intake from food only**	455.79 ± 1.87	28.09	450.69 ± 2.33 ^a^	28.54	460.41 ± 2.86 ^b^	27.68 **	*p* = 0.009
**Plain Water**	805.66 ± 8.91	48.36	767.32 ± 11.22 ^a^	47.61	840.49 ± 13.39 ^b^	49.05 *	*p* < 0.001
Bottled water	685.54 ± 6.98	41.75	671.51 ± 8.67 ^a^	42.15	698.29 ± 10.72 ^b^	41.39	*p* = 0.053
Tap water	96.36 ± 6.75	5.62	81.26 ± 8.67 ^a^	4.89	110.08 ± 10.15 ^b^	6.29	*p* = 0.031
**Water intake from all beverages (excluding plain water)**	1194.92 ± 8.91	71.60	1143.53 ± 14.17 ^a^	70.85	1241.62 ± 17.47 ^b^	72.28	*p* < 0.001
**Milk and milk alternatives**	57.72 ± 0.90	3.54	59.59 ± 1.32 ^a^	3.75	56.02 ± 1.22 ^b^	3.34 **	*p* = 0.048
Milk	25.62 ± 0.81	1.56	26.75 ± 1.20 ^a^	1.67	24.59 ± 1.09 ^a^	1.45 *	*p* = 0.182
Milk Alternatives ǂ	32.10 ± 0.32	1.98	32.84 ± 0.44 ^a^	2.08	31.43 ± 0.45 ^b^	1.89 **	*p* = 0.026
**Sodas**	155.68 ± 5.35	9.25	132.14 ± 7.15 ^a^	8.09	177.06 ± 7.74 ^b^	10.31 **	*p* < 0.001
Regular	142.61 ± 4.81	8.50	114.04 ± 5.15 ^a^	7.06	168.68 ± 7.66 ^b^	9.80 **	*p* < 0.001
Diet	13.01 ± 2.83	0.75	18.10 ± 5.50 ^a^	1.03	8.38 ± 2.05 ^a^	0.50	*p* = 0.098
**Fruit Juice**	160.77 ± 1.39	9.91	163.20 ± 2.21 ^a^	10.33	158.55 ± 1.72 ^a^	9.53 **	*p* = 0.094
Fresh Fruit Juice (100% Natural)	69.15 ± 0.20	4.29	69.02 ± 0.28 ^a^	4.39	69.23 ± 0.28 ^a^	4.19 **	*p* = 0.603
Bottled Fruit Juice (with sugar)/Fruit Drink	85.18 ± 0.78	5.25	85.90 ± 1.11 ^a^	5.43	84.52 ± 1.09 ^a^	5.08 **	*p* = 0.379
Bottled Fruit Juice (without sugar)	6.46 ± 1.18	0.38	8.28 ± 2.07 ^a^	0.51	4.80 ± 1.25 ^a^	0.27	*p* = 0.152
**Vegetable Juice**	2.89 ± 0.91	0.17	4.33 ± 1.80 ^a^	0.27	1.58 ± 0.60 ^a^	0.09	*p* = 0.149
Fresh Vegetable Juice	1.26 ± 0.31	0.07	1.65 ± 0.52 ^a^	0.10	0.91 ± 0.35 ^a^	0.05	*p* = 0.232
Bottled Vegetable Juice	1.63 ± 0.86	0.10	2.67 ± 1.73 ^a^	0.16	0.68 ± 0.50 ^a^	0.04	*p* = 0.268
**Hot Beverages**	57.06 ± 2.04	3.44	52.14 ± 2.80 ^a^	3.26	61.53 ± 2.93 ^b^	3.61	*p* = 0.021
**Sports and Energy Drinks**	1.84 ± 0.62	0.10	0.81 ± 0.53 ^a^	0.05	2.77 ± 1.07 ^a^	0.14	*p* = 0.103
Sports Drinks	0.85 ± 0.49	0.05	0.47 ± 0.47 ^a^	0.03	1.20 ± 0.84 ^a^	0.06	*p* = 0.463
Energy Drinks	0.99 ± 0.32	0.05	0.34 ± 0.24 ^a^	0.02	1.58 ± 0.57 ^b^	0.08	*p* = 0.047
**Alcoholic beverages**	0.51 ± 0.21	0.03	0.02 ± 0.02 ^a^	0.00	0.95 ± 0.40 ^b^	0.06 *	*p* = 0.019

^a,b^ Statistical comparisons were conducted between age groups for the means of water intake from each beverage type using independent samples *t*-test. Mean estimates within a row with different superscript letters were significantly different at *p* < 0.05. ǂ Milk alternatives include milk-shakes, yoghurts (plain and flavored), and hot chocolate. † Statistical comparisons were conducted between age groups for the means of contribution of different beverages to total water intake (% TWI) using independent samples T-test; * *p* ≤ 0.05; ** *p* ≤ 0.001.

**Table 3:** Intakes (mL/day) of water from specific beverages * by socioeconomic ** and physical activity characteristics in a representative sample of Lebanese children aged 4–13 years † (*n* = 752).

	Bottled Water	Tap Water	Milk and Milk Alternatives	Regular Soft Drinks	Fresh Fruit Juice	Fresh Veg. Juice	Energy Drinks
	685.54 ± 6.98	96.36 ± 6.75	57.72 ± 0.90	142.67 ± 4.81	69.13 ± 0.20	1.26 ± 0.31	0.99 ± 0.32
**Father’s educational level**							
Intermediate or less	670.04 ± 9.09 ^a^	115.67 ± 9.65 ^a^	56.48 ± 1.18 ^a^	155.95 ± 6.20 ^a^	69.10 ± 0.26 ^a^	1.41 ± 0.47 ^a^	0.92 ± 0.40 ^a^
High school/technical diploma	699.88 ± 14.23 ^a,b^	82.80 ± 12.37 ^a,b^	58.98 ± 1.68 ^a^	125.42 ± 9.16 ^b^	68.95 ± 0.42 ^a^	1.25 ± 0.48 ^a^	1.63 ± 0.83 ^a^
University Degree or more	722.11 ± 16.45 ^b^	41.01 ± 9.12 ^b^	60.30 ± 2.53 ^a^	106.77 ± 9.95 ^b,c^	69.53 ± 0.50 ^a^	0.76 ± 0.44 ^a^	0.11 ± 0.08 ^a^
**Mother’s educational level**							
Intermediate or less	653.90 ± 9.453 ^a^	122.48 ± 10.76 ^a^	53.28 ± 1.00 ^a^	168.61 ± 8.00 ^a^	68.87 ± 0.30 ^a^	0.92 ± 0.33 ^a^	1.95 ± 0.66 ^a^
High school/technical diploma	714.72 ± 13.68 ^b^	82.44 ± 11.64 ^b^	62.03 ± 1.95 ^b^	125.89 ± 7.36 ^b^	69.31 ± 0.34 ^a^	1.06 ± 0.47 ^a^	0.04 ± 0.03 ^b^
University Degree or more	715.57 ± 14.52 ^b,c^	57.33 ± 10.77 ^b,c^	61.64 ± 2.14 ^b,c^	108.04 ± 7.19 ^b,c^	69.48 ± 0.44 ^a^	2.30 ± 1.02 ^a^	0.13 ± 0.10 ^a,b^
**Monthly family income (LBP)**							
≤1,000,000	647.53 ± 10.90 ^a^	144.24 ± 12.73 ^a^	54.55 ± 1.21 ^a^	162.71 ± 8.15 ^a^	68.80 ± 0.31 ^a^	0.87 ± 0.47 ^a^	0.79 ± 0.34 ^a^
1,000,000–1,999,999	698.20 ± 10.88 ^b^	69.43 ± 9.15 ^b^	59.39 ± 1.65 ^b^	135.02 ± 6.56 ^b^	69.41 ± 0.31 ^a^	1.32 ± 0.43 ^a^	0.76 ± 0.42 ^a^
≥2,000,000	743.9447 ± 15.53 ^c^	48.09 ± 9.49 ^b,c^	59.72 ± 1.92 ^a,b^	116.72 ± 11.21 ^b,c^	69.48 ± 0.52 ^a^	2.04 ± 0.87 ^a^	1.86 ± 1.20 ^a^
**Crowding index (person/room)**							
<2	696.62 ± 8.40 ^a^	85.66 ± 7.30 ^a^	58.71 ± 1.09 ^a^	137.07 ± 5.79 ^a^	69.52 ± 0.24 ^a^	1.55 ± 0.42 ^a^	0.87 ± 0.37 ^a^
≥2	657.64 ± 12.49 ^b^	123.56 ± 14.89 ^b^	54.91 ± 1.55 ^a^	156.28 ± 8.56 ^a^	68.20 ± 0.35 ^b^	0.56 ± 0.23 ^b^	1.29 ± 0.63 ^a^
**Level of physical activity**							
Active	691.14 ± 9.42 ^a^	99.56 ± 8.82 ^a^	58.13 ± 1.21 ^a^	155.08 ± 6.43 ^a^	69.85 ± 0.25 ^a^	1.41 ± 0.46 ^a^	1.44 ± 0.53 ^a^
Inactive	677.91 ± 10.37 ^a^	91.98 ± 10.48 ^a^	57.16 ± 1.35 ^a^	125.72 ± 7.14 ^b^	68.15 ± 0.32 ^b^	1.10 ± 0.36 ^a^	0.37 ± 0.23 ^a^

* Analyses were carried for the remaining beverages (fruit juice with sugar (fruit drinks), fruit juice without sugar, bottled vegetable juice, diet soft drinks, sports drinks, alcoholic beverages, and hot beverages) but the results were not presented in this table given the lack of significant associations with physical activity or any SES variable. ** Analyses were conducted for father and mother’s employment status but the results were not included in this table since these variables were not significantly associated with water intakes from any beverage. † Analyses were carried for anthropometric variables (including BMI status, and WHtR) in relation to water intakes from beverage sources, but the results were not included in the table due to lack of significant associations. ^a,b,c^ Statistical comparisons are made within each socioeconomic group and within physical activity levels based on independent samples *t*-test or ANOVA test with Bonferroni-adjustment. Mean estimates with different superscript letters were significantly different at *p* < 0.05.

**Table 4:** Energy Intake (kcal/day) from different beverage groups and from all solid foods and their contribution to total energy intake by age group; children aged 4–13 years, Lebanon (*n* = 752).

		Total		4–8 Years 183 (46.1%)		9–13 Years 214 (53.9%)		*p*-Value
	Number of Consumers *n* (%)	Mean ± SE	% of Total EI	Mean ± SE	% of Total EI	Mean ± SE	% of Total EI †	
**Total energy intake from all foods and beverages**	752 (100)	1924.66 ± 7.45	100	1899.34 ± 8.80 ^a^	100	1947.67 ± 11.66 ^b^	100	0.001
**Energy intake from foods only**	752 (100)	1741.56 ± 6.34	90.54	1720.68 ± 7.71 ^a^	90.62	1760.54 ± 9.77 ^b^	90.47	0.001
**Energy intake from caloric beverages**	752 (100)	181.16 ± 1.49	9.36	176.76 ± 1.76 ^a^	9.27	185.17 ± 2.33 ^b^	9.44	0.004
**Milk and milk alternatives**	752 (100)	56.15 ± 1.24	2.92	58.28 ± 1.94 ^a^	3.07	54.20 ± 1.57 ^a^	2.79 *	0.080
Milk	752 (100)	32.25 ± 1.18	1.67	34.45 ± 1.88 ^a^	1.80	30.25 ± 1.46 ^a^	1.55 *	0.079
Milk alternatives	752 (100)	23.90 ± 0.23	1.25	23.84 ± 0.29 ^a^	1.26	23.95 ± 0.35 ^a^	1.24	0.972
**Sodas**	752 (100)	60.22 ± 1.98	3.06	48.54 ± 2.12 ^a^	2.51	70.83 ± 3.16 ^b^	3.56 **	<0.001
Regular	752 (100)	59.96 ± 1.98	3.05	48.18 ± 2.13 ^a^	2.49	70.67 ± 3.16 ^b^	3.55 **	<0.001
Diet	68 (9.04)	0.26 ± 0.06	0.01	0.36 ± 0.11 ^a^	0.02	0.17 ± 0.04 ^a^	0.01	0.098
**Fruit Juice**	752 (100)	71.34 ± 0.82	3.71	73.01 ± 1.30 ^a^	3.85	69.82 ± 1.02 ^a^	3.58 **	0.052
Fresh fruit juice (100% natural)	752 (100)	15.24 ± 0.14	0.80	15.48 ± 0.22 ^a^	0.82	15.02 ± 0.18 ^a^	0.78 *	0.096
Bottled fruit juice (with sugar)/fruit drink	752 (100)	52.51 ± 0.48	2.72	52.92 ± 0.68 ^a^	2.78	52.13 ± 0.67 ^a^	2.67 *	0.406
Bottled fruit juice (without sugar)	41 (5.45)	3.59 ± 0.66	0.18	4.61 ± 1.15 ^a^	0.24	2.67 ± 0.69 ^a^	0.13	0.152
**Vegetable juice**	50 (6.65)	0.56 ± 0.18	0.03	0.84 ± 0.36 ^a^	0.05	0.30 ± 0.12 ^a^	0.02	0.157
Fresh vegetable juice	42 (5.59)	0.23 ± 0.06	0.01	0.30 ± 0.09 ^a^	0.02	0.16 ± 0.06 ^a^	0.01	0.232
Bottled vegetable juice	8 (1.06)	0.33 ± 0.18	0.02	0.54 ± 0.35 ^a^	0.03	0.14 ± 0.10 ^a^	0.01	0.268
**Hot Beverages**	752 (100)	0.70 ± 0.01	0.037	0.67 ± 0.01 ^a^	0.03	0.72 ± 0.01 ^b^	0.04 *	0.001
**Sports and energy drinks**	21 (2.79)	0.75 ± 0.23	0.04	0.31 ± 0.18 ^a^	0.02	1.15 ± 0.40 ^a^	0.06	0.059
Sports drinks	6 (0.80)	0.25 ± 0.14	0.01	0.14 ± 0.14 ^a^	0.01	0.35 ± 0.24 ^a^	0.02	0.463
Energy drinks	18 (2.39)	0.50 ± 0.16	0.02	0.17 ± 0.12 ^a^	0.01	0.80 ± 0.29 ^b^	0.04 *	0.047
**Alcoholic beverages**	13 (1.73)	0.48 ± 0.25	0.02	0.01 ± 0.01 ^a^	0.00	0.90 ± 0.48 ^a^	0.04 *	0.064

^a,b^ Statistical comparisons were carried out between age groups for the means of energy intake from each beverage type using independent samples *t*-test. Mean estimates within a row with different superscript letters were significantly different at *p* < 0.05. † Statistical comparisons were conducted between age groups for the means of contribution of different beverages to total energy intake (% EI) using independent samples T-test. * *p* ≤ 0.05; ** *p* ≤ 0.001.

**Table 5:** Patterns of water and beverage consumption amongst 4–13-year-old Lebanese children as compared to data reported by other studies on the same age group.

Present Study (Lebanon)	France ^a^		USA ^b^		Mexico ^c^		Present Study (Lebanon)	
	4–8 years	9–13 years	4–8 years	9–13 years	4–8 years	9–13 years	4–8 years	9–13 years
**Total water intake (TWI) (mL/day)**	1233	1416	1447	1711	1426.8	1658.4	1600.63	1697.68
**Plain water intake (mL/day)**	407.6	498.2	364.9	496.1	350.2	426.9	767.32	840.49
**Contribution of plain water to TWI (%)**	33	35	25.2	29	24.5	25.7	47.61	49.05
**Water intake from foods (mL/day)**	492.3	555.5	431.4	457.5	504.8	597.2	450.69	460.41
**Contribution of foods to TWI (%)**	40	39.2%	29.8	26.7	35.4	36.0	28.5	27.7
**Water intake from all beverages (mL/day)**	740.7	860.6	1015.4	1254	922.1	1061.2	1143.53	1241.62
**Contribution of all beverages to TWI (%)**	60.1	60.7	70.2	73.3	64.6	64.0	70.85	72.28
**Water intake from all beverages (excluding plain water) (mL/day)**	333.3	362.4	M: 678 ^d^	M: 814 ^d^	571.9 ^d^	634.3 ^d^	376.2	401.1
			F: 621 ^d^	F: 702 ^d^				
**Contribution of all beverages (excluding plain water) to TWI (%)**	27.0	25.6	M: 46.9	M: 47.6	40.1	38.2	23.5	23.6
			F: 43.0	F: 41.0				
**Beverages with the highest contribution to TWI**	4–13 years: Plain water (33%–35%)		Plain Water: 25.2%	Plain Water (29%)	Plain water: (24.5%)	Plain water (25.7%)	Plain water (47.6%)	Plain water (49.05%)
	Milk (13.2%)		Milk (20.4%)	Milk (15.7%)	Fruit water (8.8%)	Fruit water (8.6%)	Fruit juice (10.3%)	Soda: 10.3%
	Fruit juice (5.5%)		Fruit drink (7.9%)	Soda (12%)	Milk (5.5%)	Soda (7%)	Soda (8.1%)	Fruit juice (9.5%)
Proportion of children not meeting AI *	89	Boys: 90	75	Boys: 85	71	Boys: 83	74.0	Boys: 92.1
		Girls: 93		Girls: 83		Girls: 81		Girls: 97.8
Shortfall compared to AI (mL/day) *	367	Boys: 594	253	Boys: 633	273	Boys: 668	99	Boys: 592
		Girls: 587		Girls: 444		Girls: 516		Girls: 533
**Water to energy ratio**	4–13 years: Boys: 0.75;		0.85	Boys: 0.88	0.84	Boys: 0.82	0.84	0.87
	Girls: 0.77			Girls: 0.95		Girls: 0.84		
**Contribution of caloric beverages to total EI (%)**	11.5	10.1	19.5	18	19.8	17.5	9.27	9.44

EI: Energy Intake; * USA and Mexico: the AI is based on the IOM; France: the AI is based on EFSA; ^a^ Vieux et al., 2016 [17]; ^b^ Drewnowski et al., 2013 [9]; ^c^ Piernas, C, Barquera, S, and Popkin, BM, 2014 [11]; ^d^ Calculated from data reported by the studies [9,11].
